# A genome scan for selection signatures comparing farmed Atlantic salmon with two wild populations: Testing colocalization among outlier markers, candidate genes, and quantitative trait loci for production traits

**DOI:** 10.1111/eva.12450

**Published:** 2016-12-29

**Authors:** Lei Liu, Keng Pee Ang, J. A. K. Elliott, Matthew Peter Kent, Sigbjørn Lien, Danielle MacDonald, Elizabeth Grace Boulding

**Affiliations:** ^1^ Department of Integrative BiologyUniversity of GuelphGuelphONCanada; ^2^ Cooke Aquaculture Inc.Blacks HarbourNBCanada; ^3^ Department of Animal and Aquacultural Sciences (IHA)Center for Integrative Genetics (CIGENE)Norwegian University of Life SciencesÅsNorway; ^4^ Saint Andrews Biological StationDepartment of Fisheries and Oceans CanadaSaint AndrewsNBCanada; ^5^ Present address: School of Marine SciencesNingbo UniversityNingboChina

**Keywords:** artificial selection, Atlantic salmon, candidate genes, continent of origin, domestication selection, outlier tests, population structure, SNP

## Abstract

Comparative genome scans can be used to identify chromosome regions, but not traits, that are putatively under selection. Identification of targeted traits may be more likely in recently domesticated populations under strong artificial selection for increased production. We used a North American Atlantic salmon 6K SNP dataset to locate genome regions of an aquaculture strain (Saint John River) that were highly diverged from that of its putative wild founder population (Tobique River). First, admixed individuals with partial European ancestry were detected using STRUCTURE and removed from the dataset. Outlier loci were then identified as those showing extreme differentiation between the aquaculture population and the founder population. All Arlequin methods identified an overlapping subset of 17 outlier loci, three of which were also identified by BayeScan. Many outlier loci were near candidate genes and some were near published quantitative trait loci (QTLs) for growth, appetite, maturity, or disease resistance. Parallel comparisons using a wild, nonfounder population (Stewiacke River) yielded only one overlapping outlier locus as well as a known maturity QTL. We conclude that genome scans comparing a recently domesticated strain with its wild founder population can facilitate identification of candidate genes for traits known to have been under strong artificial selection.

## 1 Introduction

Animal domestication is one of the most important practical applications of evolutionary theory in human history. Domestication is the evolutionary process of genetic adaptation of wild animal populations to environmental conditions created deliberately or accidently by humans. This process can involve changes in morphological, physiological, behavioral, and life history traits (Mignon‐Grasteau et al., 2005) and began at least 15,000 YBP, when dogs were domesticated to facilitate hunting or to guard human settlements (Axelsson et al., 2013; Braastad & Bakken, 2002; Frantz et al., 2016). Subsequently, from 8,000 to 10,000 YBP, pigs, sheep, goats and cattle were domesticated as a source of food (Craig, 1981). In fish, domestication may have begun in artificial ponds that were constructed more than 3,615 YBP (Balon, 2004; Jiang, Li, Ming, & Bao, 2012).

Domestication can leave detectable signatures of selection within the genomes of agricultural species because of strong artificial selection for specific phenotypes that increase yield in a controlled farm environment. Comparison of the genomes of domesticated species to their wild founder populations can help identify the genes underlying differentially selected traits, thereby advancing a fundamental goal of evolutionary biology (Stinchcombe & Hoekstra, 2008). Population genomics approaches have been used to support the hypothesis of artificial selection by humans over the past millennium causing allele frequency changes at major loci that determine cob size in maize (Vigouroux et al., 2002), muscle growth in pigs (Van Laere et al., 2003), and coat color in domestic mammals (Cieslak, Reissmann, Hofreiter, & Ludwig, 2011). Motivation behind genome scans for selection signatures lies in the possibility of finding DNA markers associated with traits of economic interest that can be used for marker‐assisted selection (López, Neira, & Yáñez, 2015; Yáñez, Houston, & Newman, 2014).

Two scenarios can theoretically arise when a domestic population is founded from a wild population and then subjected to strong artificial selection for a value of a trait that is largely determined by a major locus: (i) a hard sweep where a new favorable mutation at the major locus rapidly becomes fixed, initially resulting in a single long haplotype block surrounding the mutation that is well differentiated from the wild population along its entire length, (ii) a soft sweep where the frequency of a pre‐existing favorable allele at the major locus increases along with all the multiple original haplotypes containing it; the latter results in short haplotype blocks immediately surrounding the locus that are differentiated from the wild population but preserves pre‐existing variation in more distant regions (López et al., 2015). Genome scans support the existence of long haplotype blocks in domestic chickens where regions of low heterozygosity containing previously described candidate genes for laying traits that can span several Megabases (Mb) suggesting recent hard sweeps (Qanbari et al., 2012). Soft sweeps characterized by narrow divergent haplotype blocks were more common than hard sweeps characterized by wide divergent haplotype blocks in a population of maize that had undergone strong positive directional selection for ear number for 30 generations (Beissinger et al., 2014). In practice, these two scenarios represent a continuum with the long haplotype blocks of the first becoming shorter and harder to detect over millennia unless they are in a nonrecombining region of the genome (Ai et al., 2015).

The increased availability of high‐resolution SNP datasets and genome resequencing datasets for livestock species has permitted genome scans of livestock populations to be compared with those of their wild ancestral population(s). Changes in the genomes of different breeds of domesticated pigs detected by comparing SNP allele frequencies with those of ancestral wild boars are attributed to nearby genes under selection (Ai et al., 2015; Li et al., 2013). Similar studies have been published in cattle (Hayes et al., 2008; The Bovine HapMap Consortium 2009) and sheep (Chessa et al., 2009).

Statistical methods are being developed to identify “outlier” loci that have either higher or lower differentiation in allele frequencies among populations than expected under models without selection (Beaumont & Balding, 2004; Beaumont & Nichols, 1996; Excoffier, Hofer, & Foll, 2009; Foll & Gaggiotti, 2008). Most methods measure genetic differentiation among populations using *F*_ST,_ a standardized measure of divergence among populations that can be calculated separately for each genetic locus (Weir & Cockerham, 1984; Wright, 1965). Markers showing higher than expected *F*_ST_ values are identified as outliers putatively under population‐specific directional selection, whereas those with lower than expected *F*_ST_ values are identified as outliers putatively under balancing selection (Cavalli‐Sforza, 1966; Lewontin & Krakauer, 1973; Strasburg et al., 2012). Outlier analyses (Beaumont & Nichols, 1996) assume that the two or more populations being compared are each monophyletic in origin. To satisfy this assumption, any sampled individuals that are hybrids between highly divergent strains or subspecies should be removed from the dataset before doing the analysis. Hybrid or admixed individuals can often be detected using software such as STRUCTURE first to estimate the number of founder population clusters present in a dataset; the average multilocus allele frequencies characteristic of each cluster can then be used to identify admixed individuals (Falush, Stephens, & Pritchard, 2003; Hubisz, Falush, Stephens, & Pritchard, 2009; Pritchard, Stephens, & Donnelly, 2000).

Signatures of selection within the genomes of Atlantic salmon may sometimes be easier to detect than those in livestock because artificial selection for economically important traits has only been implemented for 5 to 15 generations (Gjedrem, Gjoen, & Gjerde, 1991; Quinton, McMillan, & Glebe, 2005; Wood, Anutha, & Peschken, 1990) rather than 5,000 generations (Craig, 1981). Firstly, because of the low number of generations, samples from putative wild founder populations that that have not experienced large changes in allele frequencies from genetic bottlenecks are more likely to be available. Secondly, 5–15 generations is insufficient time for recombination to have reduced the size of long haplotype blocks resulting from hard sweeps (Sabeti et al., 2002) making outlier loci easier to detect with a low density of markers. Finally, realistic simulations of a single locus trait that is under moderately strong selection (s > 0.25) in the aquaculture population but not in the wild founder population for fewer than 10 generations show that the statistical power to detect the divergence in allele frequencies is high (ß > 0.8) (Karlsson & Moen, 2010).

In contrast, the similar selection differentials applied to classic quantitative traits like growth are assumed to cause only minor changes in allele frequencies (Falconer & Mackay, 1996) that would be undetectable using outlier locus methodology (Wellenreuther & Hansson, 2016), but see Fontanesi et al. (2015). Both single locus and quantitative traits might show rapid genetic divergence between aquaculture strains and wild populations of Atlantic salmon. Traits that may change in aquaculture strains as a direct response to artificial selection include increased growth rate, later adult maturity, redder flesh color, and better taste (Rye, Gjerde, & Gjedrem, 2010). Traits that may change as an indirect response to selection for increased growth rate under hatchery conditions include increased levels of aggression, increased boldness, altered parasite or disease resistance, increased appetite, and higher food conversion efficiency (Araki & Schmid, 2010; Bekkevold, Hansen, & Nielsen, 2006).

Application of genome scans to Atlantic salmon populations has been facilitated by the greatly increased availability of SNPs for Atlantic salmon (Houston et al., 2014; Lien et al., 2011) and the publication of the genome (Lien et al., 2016). Recent outlier locus studies of salmonid fishes have primarily compared wild populations at different spatial scales (Freamo, O'Reilly, Berg, Lien, & Boulding, 2011; Vasemägi & Primmer, 2005) or have correlated outlier loci of wild populations with specific environmental variables (Bourret, Kent, et al., 2013; Bourret, Dionne, Kent, Lien, & Bernatchez, 2013; Narum, Campbell, Kozfkay, & Meyer, 2010; Perrier, Bourret, Kent, & Bernatchez et al., 2013). Of more relevance to Atlantic salmon domestication are four outlier studies comparing aquaculture strains with wild populations from the same region as their putative founder population(s) (Gutierrez, Yáñez, & Davidson, 2016; Karlsson, Moen, Lien, Glover, & Hindar, 2011; Mäkinen, Vasemägi, McGinnity, Cross, & Primmer, 2015; Vasemägi, Nilsson, & Primmer, 2005).

We hypothesized that outlier analysis that compared the genomes of recently created North American aquaculture populations of Atlantic salmon from the Saint John River (SJR), with that of their putative wild founder population from the Tobique River, would be likely to identify genome regions underlying traits known to be under strong artificial selection in the hatchery environment. First, STRUCTURE was used to identify admixed individuals (Bradbury et al., 2015; Pritchard et al., 2016) with partial European ancestry so that they could be removed from the dataset. Second, two outlier locus detection programs (Arlequin 3.5 and BayeScan 2.1) were used to compare the putative founder population to four nonoverlapping year classes of the aquaculture population. Parallel analyses comparing the aquaculture population with a nearby nonfounder wild population from the Stewiacke River were conducted to determine whether an overlapping set of outlier loci would be discovered. Thirdly, we located the chromosome position of each outlier locus on linkage maps and compared its position with that of previously published: a) outlier loci, b) candidate genes, and c) QTLs for growth, life history, and immune traits. This allowed us to identify the genome regions, as well as some putative candidate genes, that have responded to either deliberate artificial selection for growth and late maturity or to accidental selection exerted by the hatchery environment.

## 2 Materials and methods

### 2.1 Sampling strategy and SNP genotyping

Atlantic salmon belonging to eight putative populations and one hybrid population were sampled between 2006 and 2012 (Table 1). The Canadian populations included two generations from four independent year classes (hereafter “populations”) of the SJR aquaculture strain (AQUA). It also included 98 hatchery‐spawned adults captured as wild smolts from the Tobique River (TOB_WILD), which is an upper tributary of the SJR, and 100 hatchery‐spawned “wild” adults from the Stewiacke River (STW_WILD) as part of the live gene‐banking program (O'Reilly & Doyle, 2007).

**Table 1:** Atlantic salmon (*Salmo salar* L.) samples analyzed in the present study

Group	Population name^a^	Gen (strain)^b^	Abbreviation	Sample size
Aquacultural Group	2008–2009 Parents & Offspring	6 (84JC)	2009PO_AQUA	134
	2009–2010 Parents & Grandparents	5 (89JC)	2010PG_AQUA	250
	2010–2011 Parents & Grandparents	5 (90JC)	2011PG_AQUA	268
	2010–2011N Parents & Grandparents^c^	5 (90JC)	2011PGN_AQUA	96
	2011–2012 Parents & Grandparents	6 (87JC)	2012PG_AQUA	191
	Mowi (EU)^d^	10	MOWI	8
	Hybrids (AQUA × MOWI)^e^	–	Hybrids	10
Wild Founder	Tobique River Wild Population^f^	1	TOB_WILD	98
Wild Outgroup	Stewiacke River Wild Population^g^	1	STW_WILD	100

a All are North American (NA) subspecies of *Salmo salar* unless otherwise indicated. Each population of the SJR AQUA strains was primarily derived from a single year class of fish from the Mactaquac Biodiversity Facility (see text of Methods). Historically, the 4‐year classes of the SJR AQUA strain were spawned on a 4‐year cycle so that the offspring of year 1 of a cycle were the parents of year 1 of the next cycle (J. A. K. Elliott, pers. obs.).

b Number of generations in captivity (year strain founded from fish returning to the Mactaquac Dam).

c Relatives of the broodstock in the main 2010–2011 breeding nucleus.

d European (EU) subspecies of *Salmo salar*. The founder fish for the Mowi strain were collected from the Voss River, Norway, and surrounding areas in 1964 (Ferguson et al., 2007).

e F1 hybrids (2001) and F1 backcrosses (2005) between NA and EU (Mowi) subspecies of *Salmo salar* (Boulding et al., 2008).

f The sampled “wild‐exposed two‐year salmon” from single‐pair crosses were captured as smolts or presmolts from the wild by DFO technicians (i.e., caught as smolts in the rotary screw traps set lower in the Tobique River system) then reared in the Mactaquac hatchery for 2 years to maturity. Fin clips were taken during spawning in late Fall 2010 under the supervision of D.M.

g Stewiacke River reared at Coldbrook Biodiversity Facility, NS from single‐pair crosses. Fin clips were taken during spawning in late Fall 2010 under the supervision of S. Ratelle.

All AQUA populations were primarily founded from four separate year classes of Atlantic salmon that were collected by staff from the Mactaquac Biodiversity Facility (MBF) hatchery (Farmer, 1991; Friars, Bailey, & O'Flynn, 1995; Glebe, 1998; O'Flynn, Bailey, & Friars, 1999). The MBF was built in 1968 to mitigate the effects of the Mactaquac dam on the SJR. The completion of the dam in 1968 prevented salmon from swimming upriver to their historical spawning areas. Thereafter, Atlantic salmon that returned to spawn just below the Mactaquac dam were collected in a “fish lift”. The “wild” fish were transferred from the lift to a truck and released upriver while the “ranched” fish were transferred to holding tanks and spawned in the Mactaquac Biodiversity Facility. The Tobique River comprised about 60% of the original spawning habitat of the SJR tributaries above the Mactaquac dam (http://www.inter.dfo-mpo.gc.ca/Maritimes/Biodiversity-Facilities).

The subsequent 4–5 generations of each of the four separate AQUA populations were created by crossing the 50 male and the 100 female candidate broodstock with the highest estimated breeding values for saltwater growth in a paternal half‐sibling design (J. A. K Elliott, pers. obs.).

Atlantic salmon populations in the Bay of Fundy in eastern Canada can be divided into two regional groups: those in the outer Bay of Fundy (oBoF) [e.g., the Saint John River (SJR) and the Tobique River] and those in the inner Bay of Fundy (iBoF) (e.g., Stewiacke River), using evidence from microsatellite analysis (King, Kalinowski, Schill, Spidle, & Lubinski, 2001), allozyme studies (Verspoor, O'Sullivan, Arnold, Knox, & Amiro, 2002), gene expression patterns (Tymchuk, O'Reilly, Bittman, Macdonald, & Schulte, 2010), and SNPs (Freamo et al., 2011). We chose TOB_WILD as a putative founder population of AQUA; however, we acknowledge that salmon from any tributary of the SJR above the Mactaquac dam (http://atlanticsalmonfederation.org/rivers/newbrunswick.html) could potentially have contributed to AQUA (Farmer, 1991). The iBoF—STW_WILD population was chosen as a putative outgroup because it is not thought to have contributed to the SJR aquaculture strain (O'Reilly & Doyle, 2007) and because it is known to grow more slowly than the oBoF—TOB_WILD population (Culling et al., 2013). Finally, to identify individuals from AQUA populations with partial European ancestry, we genotyped a purebred, full‐sibling family of fish from the Mowi aquaculture strain (MOWI_EU) founded from the Voss River region in Norway (Ferguson et al., 2007) as well as some hybrids from documented F1 and backcrosses between MOWI_EU and AQUA (Boulding et al., 2008). This was necessary because AQUA had been reported to have some residual European ancestry due to the importation of the European subspecies into Maine (Glebe, 1998; O'Reilly, Carr, Whoriskey, & Verspoor, 2006). Atlantic salmon (*Salmo salar* L.) can be characterized as two subspecies, the “North American” and the “European” (King et al., 2007), which differ in the number of chromosomes (Brenna‐Hansen et al., 2012).

Fin clips were collected from live fish and preserved in 95% ethanol for later DNA analysis. DNA was extracted from all fin clip samples using 96‐well plate format Qiagen^™^ DNeasy blood and tissue kits, according to the protocol recommended by the manufacturer. A custom Atlantic salmon Illumina iSelect^™^ 6K bead array (Brenna‐Hansen et al., 2012; Lien et al., 2011) was used to perform SNP genotyping. Once all fish were genotyped, all genotype clusters were assigned using Illumina Genome Studio© software and subsequently checked manually. The more complex paralogous sequence variants (PSVs) and multisite variants (MSVs) were identified through visual inspection of GenomeStudio's polar coordinate graphs with signal intensity, norm R, on the *y* axis; and allele frequency, norm theta, on the *x* axis. Only diploid loci identified by GenomeStudio as (i) showing Mendelian inheritance in our population and (ii) not significantly deviating from Hardy–Weinberg equilibrium were used for subsequent analyses. Nearly all SNP loci used in our analyses had been located on a North American Atlantic salmon female linkage map (Brenna‐Hansen et al., 2012) or a European linkage map (Lien et al., 2011).

### 2.2 Population structure analysis

All individuals from the nine sampled putative populations were included in the Bayesian cluster population structure analysis with STRUCTURE 2.3.4 (Hubisz et al., 2009; Pritchard et al., 2000). To infer the optimal number of clusters, *K*, the STRUCTURE simulation results were analyzed according to the delta *K* method (Evanno, Regnaut, & Goudet, 2005). We set the *K* from 1 to 10, so that the maximum number was larger than the number of putative populations (*n* = 9), thereby avoiding inappropriate clustering due to *K* being set too small (Kalinowski, 2011). The simulations were repeated for values of *K* ranging from 1 to 10, with five repeat runs for each *K* value using the admixture model and no prior probabilities for cluster membership. For each simulation at a given *K* value, a burn‐in period of 50,000 iterations was followed by 500,000 final iterations. Finally, the graphical bar plot of membership coefficients was generated using the DISTRUCT software with the fill color of the bars indicating cluster membership (Rosenberg, 2004). Each individual fish was also given an estimated membership coefficient for each of the *K* clusters, corresponding to the fraction of its genome inferred to have ancestry in the cluster. Some individuals in our dataset were assigned to two or more clusters (or putative, genetically distinct populations) suggesting that their genotypes were admixed because of past hybridization.

### 2.3 Pairwise genetic distances among putative populations

We were primarily interested in comparing the genetic distance between TOB_WILD and the four populations of AQUA as they diverged from a common ancestral population five to six generations ago (Table 1). All genetic distance calculations were performed on a reduced dataset after individual fish that had been estimated by STRUCTURE to have more than 5% European ancestry had been removed. Pairwise comparisons of several measures of genetic distance were then estimated using Arlequin 3.5 (Excoffier & Lischer, 2010) with 10,000 permutations to determine statistical significance. We present both the pairwise *F*_ST_ values (Weir & Cockerham, 1984) calculated using Nei's mean number of pairwise differences between populations (Nei & Li, 1979) as well as Slatkin's linearized *F*_ST_ (Slatkin, 1995) as implemented in Arlequin 3.5 (Excoffier & Lischer, 2010). The latter is recognized as a good method of obtaining an approximately linear genetic distance for binary characters such as SNPs (Excoffier & Lischer, 2010).

### 2.4 Detecting loci under divergent selection in AQUA versus WILD

Methods of detecting *F*_ST_ outliers resulting from “soft sweeps” suitable for low‐density SNP chip datasets include (i) FDIST2, which compares the *F*_ST_ observed for a locus to the *F*_ST_ expected under neutrality relative to its observed heterozygosity assuming an “n‐island” model where the effective population size of all population is constant as is the pairwise population migration rate (Antao, Lopes, Lopes, Beja‐Pereira, & Luikart, 2008; Beaumont & Nichols, 1996), (ii) Arlequin v.3.5, which implements FDIST2 methodology with the addition of a hierarchical island option that assumes two different constant migration rates with the lower rate among regional groups of populations and the higher rate among populations within the same region (Excoffier et al., 2009), and (iii) BayeScan, which separately estimates the posterior probability that each locus is under selection without assuming that all effective population sizes and migration rates are equal (Beaumont & Balding, 2004; Foll & Gaggiotti, 2008). In this study, Arlequin 3.5 and BayeScan 2.1 were chosen for the outlier analysis to be a good combination to reduce type I (false positive) and type II (false negative) error rates, which have been evaluated using simulation (Lotterhos & Whitlock, 2014; Narum & Hess, 2011; Vilas, Perez‐Figueroa, & Caballero, 2012). FDIST2 typically detects more outliers than does BayeScan in empirical datasets of a few thousand SNPs (Tsumura et al., 2014). Other methods for detecting signatures of selection such as the long‐range haplotype (LRH) test (Sabeti et al., 2002) require higher resolution coverage of the genome for traits of unknown location than was possible with our 3980 SNP dataset.

All outlier locus detection analyses used the reduced dataset that omitted any individual identified by STRUCTURE as having European ancestry as this might have affected which outlier loci were detected (Gosset & Bierne, 2013). We used four different methods of comparing the TOB_WILD population with the AQUA populations to find regions of the genome with differences in SNP allele frequencies that were greater than would be expected due to genetic drift alone. Extreme differences in allele frequencies at a particular SNP locus are most parsimoniously explained from recent positive artificial selection on the AQUA population that has not experienced by TOB_WILD population as they shared a common ancestral population 5 to 6 generations earlier. We were particularly interested in finding “consistent” loci that were identified as being an outlier locus putatively under diversifying selection by more than one of the four methods.

Three of the outlier detection methods, Method 1 “nonhierarchical” using FDIST2, Method 2 “pairwise” using FDIST2, and Method 3 “hierarchical” island model, all used Arlequin 3.5's module “Detect loci under selection” (Excoffier & Lischer, 2010). We identified all SNP loci with *p* values <.01 that to be putative outlier loci under diversifying selection but took special note of outlier loci with *p* values <.001 (e.g., Johnston et al., 2014). Method 1 used a nonhierarchical (FDIST2) option of the “Detect loci under selection” module of Arlequin 3.5 to analyze the following populations: 2009PO_AQUA, 2010PG_AQUA, 2011PG_AQUA, 2011PGN_AQUA, 2012PG_AQUA, and TOB_WILD (Table 1). Method 2 was composed of six separate pairwise analyses using the nonhierarchical (FDIST2) option of Arlequin 3.5, each comparing one of the AQUA populations with the TOB_WILD population. Method 3 used the hierarchical island version of the outlier module in Arlequin 3.5 that implements a hierarchical FDIST2 (Excoffier et al., 2009). This module has a hierarchical island model that allows for lower migration rates among different “island” groups than among populations within groups. To allow estimation of its within‐group variance, the TOB_WILD population was randomly split into two random populations and placed in one group and the three largest AQUA populations (2010PG, {2011PG+2011PGN}, and 2012 PG) were placed in a second group. This allowed us to interpret *F*_CT_ as the outlier loci that differed between the aquacultural–wild comparisons rather than also including those from aquacultural–aquacultural comparisons. We classified loci with larger than average *F*_CT_ (or *F*_ST_) values that also had *p* values <.01 as being outliers potentially under directional selection.

To calculate the false discovery rate (FDR), we then input all *p*‐values from Arlequin for nonmonomorphic loci into the computer program SGoF+ (Carvajal‐Rodriguez & de Uña‐Alvarez, 2011). The previous version of this program, SGoF, calculates a multiple hypothesis testing adjustment using a sequential goodness of fit metatest that is especially designed for molecular biology applications where large numbers of tests are performed (Carvajal‐Rodríguez, de Uña‐Alvarez, & Rolán‐Alvarez, 2009). SGoF+ uses the maximum distance between a uniform distribution of *p* values and the observed distribution resulting in an improvement in the statistical power to reject the null hypothesis when it is false; it also estimates the *q* value (FDR) for each test (Carvajal‐Rodriguez & de Uña‐Alvarez, 2011).

In the pairwise Arlequin analyses that we conducted for Method 2, we noticed that the outliers were very similar between the parent and grandparent generations of an AQUA population (Appendix S3). Therefore, for Methods 1 and 3, we merged the data for the parents (P) and the grandparents (G) into a single “combined” population.

Method 4 used BayeScan, which employs a nonhierarchical Bayesian approach to detecting outlier loci (Foll & Gaggiotti, 2008). We used the following population groupings: 2009PO_AQUA, 2010PG_AQUA, 2011PG_AQUA, 2011PGN_AQUA, 2012PG_AQUA, and TOB_WILD and removed any monomorphic loci from the input data file. The parameter settings used were 10 pilot runs of 10,000 iterations, an additional burn‐in of 100,000 iterations, then 100,000 iterations with a sample size of 5,000 and a thinning interval of 20. Loci with *q*‐values <0.05 were considered outliers (where *q*‐values are the FDR as calculated by version 2.1 of BayeScan).

Method 1 and Method 3 outlier loci analyses that compared the AQUA populations to the STW_WILD population are only briefly presented in the main part of the manuscript because our primary focus was on divergence between the TOB_WILD and AQUA populations.

### 2.5 BLASTX analysis and chromosomal location of the outlier loci

Possible mechanisms of selection acting on outlier loci were investigated based on their similarity to known proteins. The translated DNA sequence that contained the outlier SNP was queried against the NCBI protein database (BLASTX). Simultaneously, we also referred to the “best hits” blast results for this SNP chip from Bourret, Dionne, et al. (2013), who set a minimal E‐value of 1 × 10^−3^ for BLASTX analysis with BLAST2GO and a minimal E‐value of 1 × 10^−10^ for gene ontology (GO) terms.

We also used BLASTN against the *Salmo salar* genome to identify the location of 17 consistent outlier SNPs on the physical map of the Atlantic salmon genome (Lien et al., 2016). To do this, we first searched “rs#” numbers of the outlier SNPs on the Reference SNP (refSNP) Cluster Report (https://www.ncbi.nlm.nih.gov/projects/SNP/) to find short DNA sequences containing the SNP that could be input into BLASTN. This allowed us to compare the position of each outlier SNP on the physical map with the position of conserved candidate genes in the growth hormone axis, gonadotropin axis, and the immune system that have recently been automatically annotated in physical map of the Atlantic salmon genome (http://salmobase.org/cgi-bin/gb2/gbrowse/salmon_GBrowse_Chr_NCBI/). We focused on locating adjacent genes but considered all genes in these categories within 5 Mb of the SNP. This was necessary because of the moderate density of the 6K SNP array in these North American populations.

### 2.6 Colocalization of outlier loci and published QTLs for growth, maturity, and domestication traits

We tested for colocalization of outlier loci and weight and maturity QTLs previously published for Atlantic salmon as has previously been done for whitefish (Rogers & Bernatchez, 2005), pea aphid (Via & West, 2008), apples (Leforestier et al., 2015). We attempted to identify outlier loci underlying traits known to have been under artificial selection by comparing their map position with that of published quantitative trait loci (QTL) for one classic quantitative trait—growth rate (Baranski, Moen, & Våge, 2010; Boulding et al., 2008; Gutierrez et al., 2012; Houston et al., 2009; Pedersen et al., 2013; Reid, Szanto, Glebe, Danzmann, & Ferguson, 2005; Tsai et al., 2015). The challenge was that, with the exception of Gutierrez et al. (2012), published QTLs for Atlantic salmon were only approximately positioned using low‐density SNP linkage maps or, in one case, using a low‐density microsatellite linkage map (Reid et al., 2005 that were positioned using Moen et al. 2008). Once a *p*‐value for a given QTL had been assigned to a SNP, subsequent QTLs for other traits from the same study would be assigned to adjacent SNPs. We then used each chromosome on the North American linkage map (Brenna‐Hansen et al., 2012) or the corresponding chromosome segments for each bin on the European linkage map (Lien et al., 2011) as a “bin”. We then determined whether each SNP in each bin: (i) was an *F*_CT_ outlier locus and (ii) was a marker with a significant *p* value for a published QTL for weight or for sexual maturity. This dataset was analyzed using a chi‐square test of association for the number of outliers and number of significant QTLs in each bin. In a second analysis, pairwise Pearson's correlation coefficients among the maximum values of four variables in each bin (−log10 (*p*‐value) for *F*_ST_, −log10 (*p*‐value) for *F*_CT_, −log10 (minimum *p*‐value) of a QTL, and the sum of significant QTLs) were calculated. Finally, a Manhattan plot was constructed to facilitate visual comparison between the positions of the outlier loci and that of the published QTLs. This used the continuous composite North American linkage map and −log10 (*p*‐values) for the QTLs from all ten datasets.

## 3 Results

### 3.1 Genotyping

We genotyped 1155 salmon from nine salmon populations on the 6K SNP chip. We first removed loci that had a minor allele frequency (MAF) that did not exceed 5% in any population and then removed loci that clustered poorly because they were products of simultaneously genotyping duplicated regions (MSVs, PSVs) (Appendices S1–S6). A total of 3980 SNPs remained in our dataset of which 3901 could be located on the North American Atlantic salmon linkage map.

### 3.2 Population structure

STRUCTURE analysis clearly showed that *K* = 2 groups gave the best fit to our dataset of nine putative populations (Fig. S1). All samples known to have European ancestry were assigned to cluster 2 and were omitted from all subsequent analysis. STRUCTURE also identified 252 AQUA individuals that had more than 5% admixture from cluster 2 (Fig. S2) and these individuals were also deleted from our dataset. This left 687 individuals from five AQUA populations, 98 individuals from the TOB_WILD population, and 100 individuals from the STW_WILD population (Table 1) to be used in genetic distance calculations and outlier loci analyses.

### 3.3 Pairwise genetic distances among sampled populations

As expected, STW_WILD from the iBoF was more distantly related to all AQUA populations (*F*_ST_ = 0.101–0.132; Table 2 above diagonal) than was TOB_WILD from the oBoF (*F*_ST_ = 0.016–0.046). Several of the AQUA populations were less related to each other (*F*_ST_ = 0.064 between 2009PO_AQUA and 2011PGN_AQUA) than they were to TOB_WILD (Table 2). Finally, STW_WILD was less differentiated from TOB_WILD (*F*_ST_ = 0.089) than from all of the AQUA populations. Pairwise comparisons were similar in rank whether Slatkin's linearized *F*_ST_ or Nei's mean number of pairwise differences was used as the standardized population distance between two populations. All pairwise population comparisons were significant at *p *< .001 (Footnote 1 of Table 2).

**Table 2:** Pairwise *F*_ST_ values: Slatkin's linearized *F*_ST_ a (above the diagonal) and Nei's mean number of pairwise differences^b^ (below the diagonal)^c^^,^ d

	2009PO_AQUA	2010PG_AQUA	2011PG_AQUA	2011PGN_AQUA	2012PG_AQUA	TOB_WILD	STW_WILD
2009PO_AQUA		0.051	0.057	0.064	0.028	0.046	0.132
2010PG_AQUA	0.049		0.034	0.003#	0.028	0.016	0.101
2011PG_AQUA	0.054	0.033		0.043	0.030	0.031	0.105
2011PGN_AQUA	0.060	0.003#	0.041		0.039	0.026	0.115
2012PG_AQUA	0.027	0.028	0.029	0.038		0.028	0.107
TOB_WILD	0.044	0.015	0.031	0.025	0.028		0.089
STW_WILD	0.116	0.092	0.095	0.103	0.096	0.082	

a Pairwise *F*_ST_ values (Weir & Cockerham, 1984) calculated as Slatkin's linearized *F*_ST_ (Slatkin, 1995) as implemented in Arlequin 3.5 (Excoffier & Lischer, 2010). All genetic distance measures are highly significant in a permutation test with 10,100 permutations at *p* = .00000 + −.0000 except for # which had *p* = .00762 + −.0009.

b Nei's mean number of pairwise differences between pairs of populations is a method of measuring genetic distance when the characters are binary (Nei & Li, 1979) as implemented in Arlequin 3.5 (Excoffier & Lischer, 2010). All genetic distance measures are highly significant in a permutation test with 10,100 permutations at *p* = .00000 + −.0000 except for # which had *p* = .00762 + −.0009.

c Full population names corresponding to these abbreviations are given in Table 1.

d All genetic distance calculations were performed on a reduced dataset after individuals shown by STRUCTURE to have European ancestry had been removed.

### 3.4 Outlier detection—TOB_WILD versus AQUA

**Figure 1:**
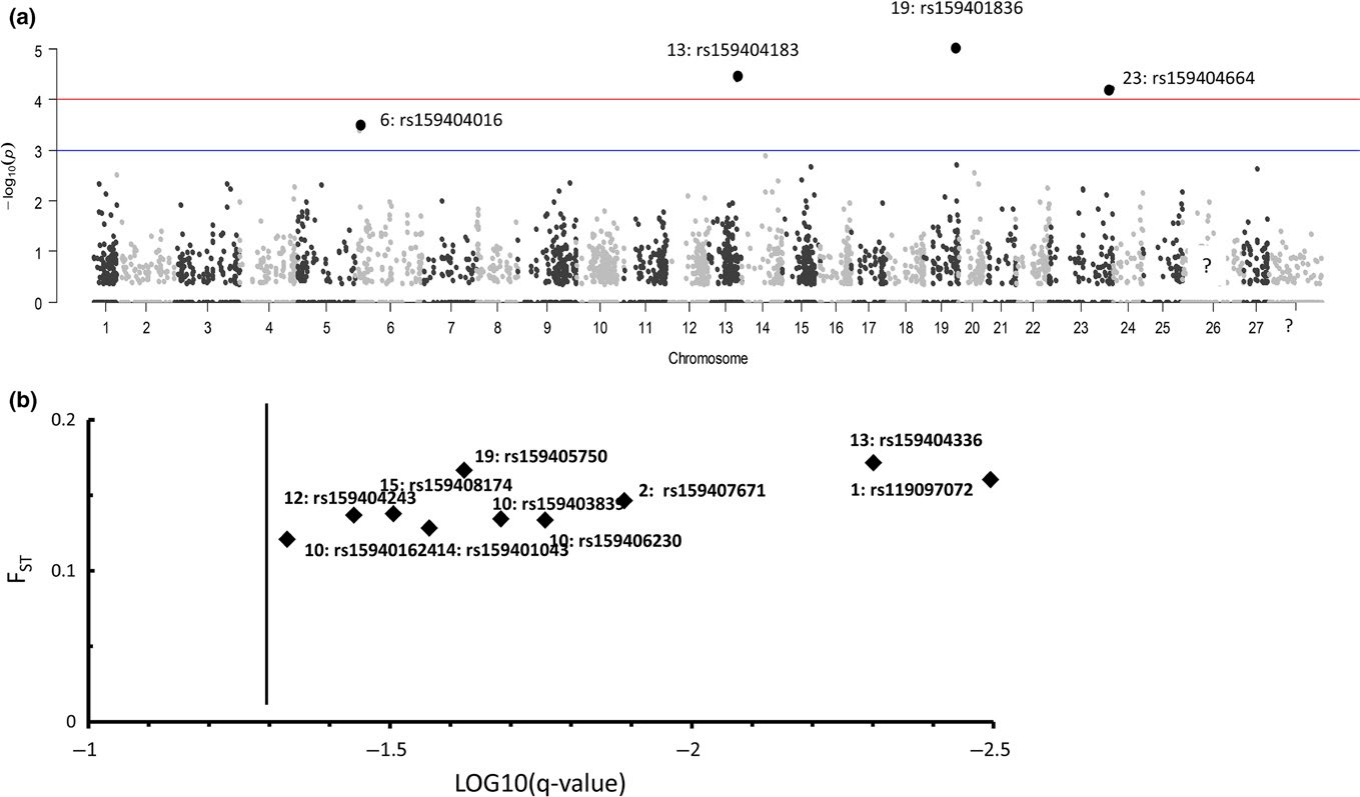
Detection of outlier loci putatively under diversifying selection within five different groups of the SJR Aquaculture strain and one wild group from the Tobique River (2009PO_AQUA, 2010PG_AQUA, 2011PG_AQUA, 2011PGN_AQUA, 2012PG_AQUA, and TOB_WILD). (a) Locus distribution on a continuous‐chromosome nonhierarchical Arlequin 3.5 analysis. The solid blue line represents the −log_10_(*p*) = 2 (*p* = .001). The solid red line corresponds to the −log_10_(*p*) = 3 (*p* = .0001) (see Appendix S1 for outlier loci numbers and official names of SNPs on chip). (b) Ten outlier loci by BayeScan 2.1 (Foll & Gaggiotti, 2008). The *F*_ST_ estimates are plotted against the false discovery rate (*q*‐values). Loci to the left of the solid black line that correspond to the *q* = 0.05 are significant outliers (see Appendix S2 for outlier loci numbers and official names of SNPs on chip)

A larger number of high‐*F*_ST_ outlier loci were identified by the Method 1 using nonhierarchical FDIST2 in Arlequin 3.5 than by Method 4 using BayeScan. The nonhierarchical analysis by Arlequin identified 37 high‐*F*_ST_ outlier loci that had *p*‐values <.01, suggesting they were putatively under positive selection in the AQUA populations. SGoF+ verified that all 37 outliers had *q*‐values <0.05. Outliers were primarily located on chromosomes 1p/23,1q, 3, 9, 12, 14, 15, and 19 (Fig. 1a; Appendix S1). In contrast, BayeScan identified only 10 outlier loci with high *F*_ST_ values and *q*‐values <0.05 (Fig. 1b; Appendix S2). Three loci were detected using both the nonhierarchical Arlequin 3.5 and BayeScan: ESTNV_24797_128, GCR_cBin4356_Ctg1_1956, and GCR_cBin15233_Ctg1_136_V2 (Appendix S1).

Method 2 identified an average of 39.2 and a total of 155 outlier loci under putatively divergent selection from the six pairwise comparisons between different AQUA populations and TOB_WILD population using FDIST2 (Appendix S3). SGoF+ verified that all 155 outliers had *q*‐values <0.05. Of the 155 loci, 66 showed semi‐parallel divergence patterns, defined as being found in at least two different AQUA versus TOB_WILD pairwise comparisons. Notably, 58 of the 66 semi‐parallel loci were found in comparisons that involved parents and grandparents that represented two generations of the same population (Appendix S3). Two “parallel” loci (GCR_cBin15233_Ctg1_136_V2 and GCR_cBin27732_Ctg1_177) were consistently identified in pairwise comparisons of each of the six AQUA populations with the TOB_WILD population (Appendix S3).

Method 3, which used the hierarchical Arlequin analysis (Table S1), found a total of 54 “high” *F*_CT_ outlier loci that were putatively under divergent selection between the AQUA and TOB_WILD populations. SGoF+ verified that all 54 outliers had *q*‐values <0.05. Of these 54 divergent *F*_CT_ outlier loci, 31 were also significantly divergent *F*_ST_ outlier loci. Both types of outlier loci were well dispersed throughout the genome (Figure 3a,b). Most strikingly, 23 of the *F*_CT_ outlier loci had not had highly significant *p*‐values (α = 0.01) for nonhierarchical *F*_ST_ analysis and thus were completely new outliers (Figure 3a; Appendix S5).

The set of *F*_ST_ outliers found by Method 1 (nonhierarchical FDIST2) and the set found by Method 3 (hierarchical) strongly overlapped (Figure 4; Appendices S1 and S5). Only six outlier loci were found solely in the analysis using Method 1 (Figure 4; Appendices S1 and S5). Both of the two parallel loci GCR_cBin15233_Ctg1_136_V2 and GCR_cBin27732_Ctg1_177 discovered by Method 2 pairwise analyses were also found by Method 1 and Method 3 analyses (Figure 4; Appendices S1 and S5).

**Figure 2:**
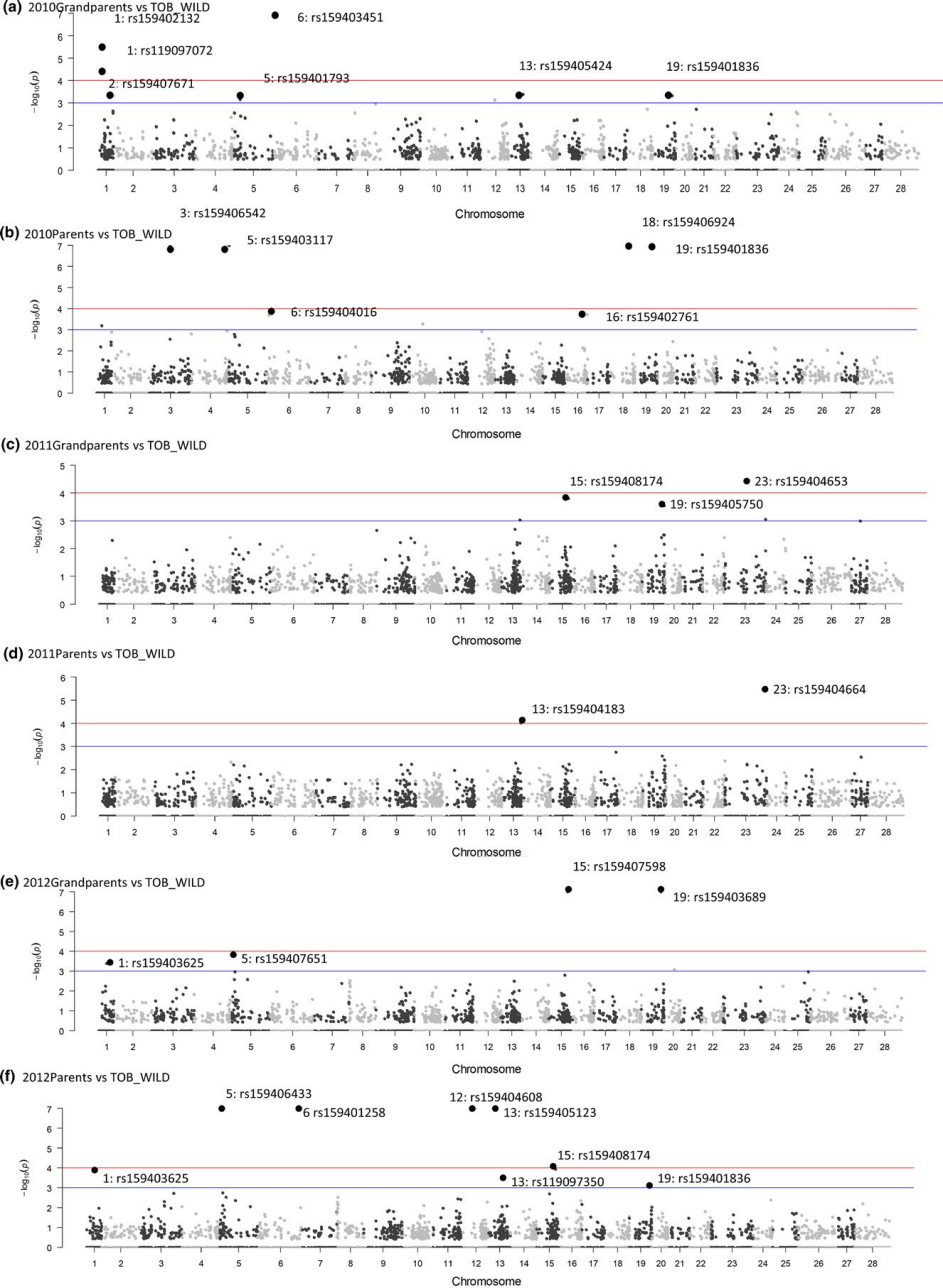
Locus distribution on a continuous chromosome for pairwise comparisons of six different generations of the AQUA population versus TOB_WILD population. The solid blue lines represent the −log_10_(*p*) = 2 (*p* = .001). The solid red lines correspond to the −log_10_(*p*) = 3 (*p* = .0001). Detailed results in Appendix S3

**Figure 3:**
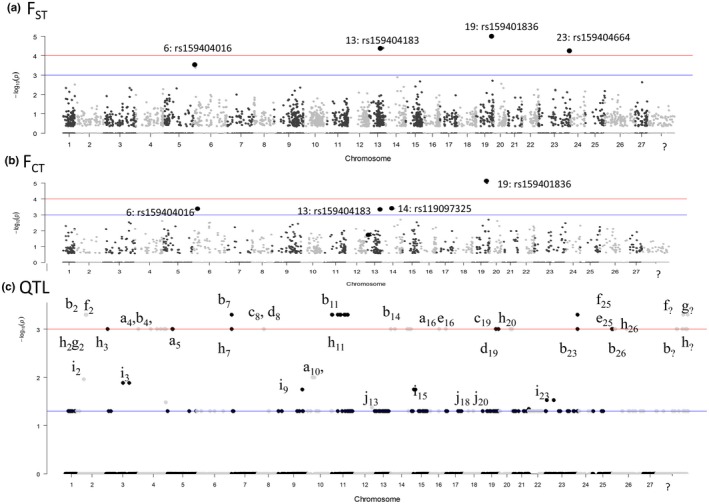
Locus distribution on a continuous chromosome from hierarchical Arlequin 3.5 analysis with the three AQUA populations {2010PG, (2011PG+2011PGN), and 2012 PG} placed into one group and the two random halves of the TOB_WILD placed in another group (Appendix S5). The line at −log_10_(*p*) = 1.5 represents *p* = .05), the line at −log_10_(*p*) = 2 represents *p* = .01, and the line at −log_10_(*p*) = 3 represents *p* = .0001. (a) *F*_ST_. (b) *F*_CT_. (c) QTL (with number showing chromosome and the letter the study “a” Baranski et al., 2010; “b” Boulding et al., 2008; “c” Gutierrez et al., 2012 males time 3, “d” QTLs from Gutierrez et al., 2012 females time 3. “e” Gutierrez et al., 2012 females time 4, “f” Gutierrez et al., 2012 Males time 4, “g” Houston et al., 2009; “h” Petersen et al. 2013, “i” Reid et al., 2005; “j” Tsai et al. 2015)

Methods 1, 2, and 3 found a total of nine unique very highly significant outlier loci with *p*‐values of <10^−4^ (Figures 1a, 2 and 3). Three of these highly significant outlier loci were classified as “consistent” because they were found by all three Arlequin Methods (Figures 1a, 2 and 3): (i) ESTNV_22997_260 (Ssa13: rs159404183) was annotated as an MHC Class II nonclassical locus, (ii) ESTNV_31210_275 (ssa01p/23: rs159404664) was annotated as palmitoyl transferase zdhhc7‐like (Table 3), and (iii) GCR_cBin27732_Ctg1_177 was one of only two parallel outlier loci that were significant for all six Method 2 pairwise comparisons in Arlequin using FDIST2 (Figure 2; Appendix S3).

**Table 3:** Protein homologies found for 23 candidate SNP loci under diversifying selection^b^ identified by nonhierarchical^c^ and hierarchical^f^ Arlequin analysis and BayeScan^d^ after outlier analysis of datasets comprising AQUA populations and the TOB_WILD population. Homologies are also shown for the 17 “consistent” loci found using all three Arlequin *F*_ST_ methods^e^. Genome position of transcript is from *Ssa* ICSASG_v2 (http://salmobase.org)

SNP Name	Nonhierarch. Arlequin^c^ *F*_ST_ (*p *< .01)	BayeScan^d^ *F*_ST_ (*q *< 0.05)	All three Arlequin *F*_ST_ methods^e^	Hierarch. Arlequin^f^ *F*_CT_ (*p *< .01)	Chr^g^ on NA Map^h^	Position NA map^h^ (cM)	Chr^g^ on EU map^h^	Position EU map^h^ (cM)	%seq. similarity	Genome position (transcript)^i^	E‐value	Matches (Gene or Protein)
ESTV_15118_153		√			1	13.7	1	94.9	70%	CIGSSA_003028.t4	1E‐75	Mitochondrial ribosomal protein s5
ESTNV_16380_81				√	3	11.5	3	0.9	99%	CIGSSA_119709.t2	2E‐125	WW domain‐containing adapter protein with coiled‐coil‐like isoform X2
GCR_cBin8095_Ctg1_103^k^					3	103.2	3	87.2	99%	CIGSSA_018951.t1	4E‐138	Protein phosphatase 1 regulatory subunit 1B‐like
ESTNV_36457_2447			√	√	4	0	4	6.1	99%	CIGSSA_024082.t6	0	Protogenin B‐like isoform X1
ESTNV_35352_77	√		√		4	101.6	4	109.9	72%	CIGSSA_024847.t1	1E‐163	RPA‐interacting protein rpain‐a
GCR_hBin33595_Ctg1_191	√		√	√	6	7.9	–	–	98%	CIGSSA_032258.t2	1E‐10	ATPase family AAA domain protein 5‐like isoform X1
ESTV_16674_300					6	63.7	–	–	100%	CIGSSA_034497.t1	3E‐14	Zona pellucida sperm‐binding protein 4‐like
ESTNV_33402_1114				√	6	67.4	6	66	100%	CIGSSA_032952.t1	0	Cleavage and polyadenylation specificity factor subunit 3
ESTNV_34703_1491			√	√	9	67.4	9	52.7	100%	CIGSSA_042894.t2	0	HCLS1‐binding protein 3
ESTNV_17881_371	√		√√		9	96.2	9	91.9	99%	XM_014214565	0	Protein phosphatase, Mg2+/Mn2+ dependent
ESTNV_24797_128	√	√	√		12	38.0	12	68.7	61.6%	CIGSSA_063980.t1	3.41E‐27	MHC class ii alpha chain
ESTNV_16810_167	√		√	√	12	70.4	12	108.3	99.8%	CIGSSA_062376.t1	0	CD34b1
ESTNV_27237_290		√			13	37.1	–	–	94%	CIGSSA_069043.t1	2.39E‐66	Orm1‐like protein 2
ESTNV_22997_260	√		√	√	13	52	13	85	97%	XM_014138527.1	0	RILP‐like protein 1 (LOC106568309), transcript variant X4,
ESTNV_28880_179	√			√	14	–	14	62	100%	CIGSSA_073641.t4	2E‐151	Probable 39S ribosomal protein L24, mitochondrial isoform X1
ESTNV_29368_168	√				15	34.9	15	44.2	83%	CIGSSA_079971.t5	2.00E‐161	Translocation associated membrane protein 2
ESTNV_34978_699			√	√	16	42.9	16	43.5	100%	CIGSSA_085177.t1	4E‐102	Glycine cleavage system H protein, mitochondrial‐like
ESTV_14714_122	√		√	√	19	61.5	19	52.7	98%	XM_014158407.1	0.0	Zinc transporter ZIP11‐like transcript variant X2, mRNA
ESTNV_31104_129	√				20^a^	–a	20	46.2	100%	CIGSSA_101213.t1	3E‐45	Rab5 gdpgtp exchange factor
ESTNV_28135_402	√			√	22	58.2	22	48.5	100%	CIGSSA_109518.t2	1E‐26	Claudin‐19 isoform X2
ESTNV_28135_544	√			√	22	58.2	22	48.5	100%	CIGSSA_109518	1E‐26	Claudin‐19 isoform X2
ESTNV_31411_621				√	22	44.5	22	35.4	100%	CIGSSA_108471.t1	1E‐92	DNA‐binding protein inhibitor ID‐1
ESTNV_31110_261^j^	√				1/23	63.3	23	13.2	91%	CIGSSA_112247.t1	2E‐26	Thrombopoietin receptor‐like
ESTNV_31110_721^j^	√				1/23	63.3	23	13.2	91%	CIGSSA_112247.t1	2E‐26	Thrombopoietin receptor‐like
ESTNV_31210_275^l^	√		√		1/23	118.1	23	51.4	87%	CIGSSA_112663.t1	0	Palmitoyltransferase ZDHHC7‐like isoform X1
ESTNV_35075_2399	√			√	25	73	25	52.7	92%	CIGSSA_117588.t3	0	Type I inositol 3,4‐bisphosphate 4‐phosphatase‐like isoform X3
ESTNV_25950_262				√	26/28	49.6	26	22.9	81%	CIGSSA_119802.t1	4E‐140	Phosphatidylinositol 3‐kinase regulatory subunit alpha‐like

a *p *< .001.

b After individuals shown by STRUCTURE to have European subspecies ancestry had been removed from the dataset.

c Non‐hierarchical Arlequin outlier analysis (Appendix S1) was done using the nonhierarchical option and included five AQUA populations: 2010PG_AQUA, (2011PG_AQUA + 2011PGN_AQUA), 2012PG_AQUA as well as the TOB_WILD population.

d BayeScan analysis (Figure 2; Appendix S2) is always nonhierarchical and included five AQUA populations: 2009PO_AQUA, 2010PG_AQUA, 2011PG_AQUA, 2011PGN_AQUA, 2012PG_AQUA, as well as the TOB_WILD population.

e Hierarchical Arlequin outlier analysis (Appendix S5) was performed using the hierarchical option. The three large populations of AQUA populations (2010PG, {2011PG + 2011PGN}, and 2012 PG) were placed in one group, and the TOB_WILD population was randomly split into two random populations (see text) and placed in a second group.

f Significant *F*_ST_ (*p *< .01) in three methods of Arlequin analysis including in at least one of the pairwise comparisons (Figure 2; Appendix S3).

g “Chr” means the chromosome number on which the outlier locus was located.

h “NA” means the position on the North American Atlantic salmon female linkage map by Brenna‐Hansen et al. (2012); “EU” means the position on the European Atlantic salmon female linkage map by Lien et al. (2011).

i Genome position is shown as the reference transcript name and number containing the SNP where possible as numbering of the base pairs may change in subsequent releases of Atlantic salmon (*Salmo salar*) genome.

“√” means the significance value is smaller than the significance level (see text) for that method.

j These two SNPs are 460 base pairs apart on in the same mRNA transcript and, consequently, were in perfect linkage disequilibrium with each other.

k This SNP is also an outlier locus for nonhierarchical and hierarchical comparisons of the Stewiacke populations with the AQUA populations.

Chromosome containing a SNP inferred from European map (Lien et al., 2011) after known translocations were accounted for (Brenna‐Hansen et al., 2012); consequently, the exact position on the North American chromosome is unknown.

l ESTNV_31210_275 codes for a protein that plays a role in follicle stimulating hormone activation of testicular Sertoli cells (Pedram et al. 2012).

**Figure 4:**
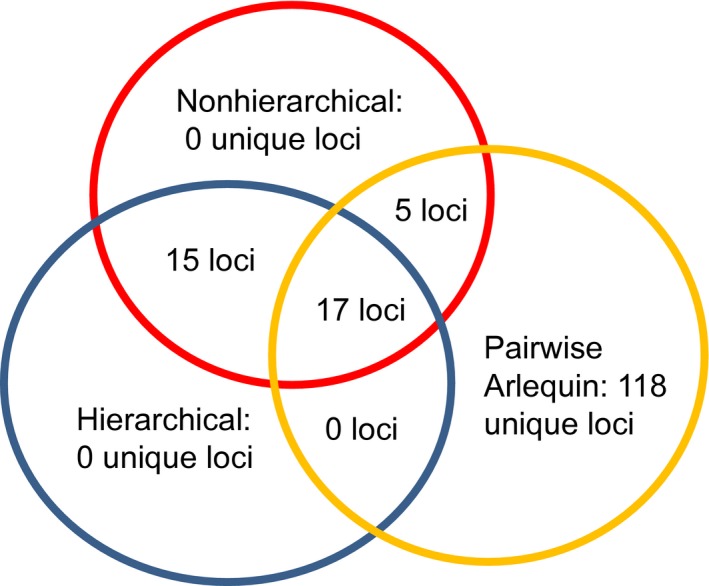
A Venn diagram with the circles representing the three different methods of outlier analysis done comparing the TOB_WILD population with the AQUA populations using Arlequin 3.5 showing: (i) the one overlapping subset of outlier loci found by all three methods of analysis, (ii) the three overlapping subsets of outlier loci that were found only by two methods of analysis, and (iii) the three subsets found exclusively by one method of analysis. The 17 loci found by all three methods are shown in Table 4

In addition to these three, a total of 17 “consistent” *F*_ST_ outlier loci were found in common among Method 1, Method 2, and Method 3 that compared the AQUA populations with the TOB_WILD population (Figure 4, Table 4). All 17 were also significant *F*_CT_ outlier loci (Appendix S5). Three of these 17 “consistent” outlier loci were also found by BayeScan (GCR_cBin15233_Ctg1_136_V2, ESTNV_24797_128, and GCR_cBin4356_Ctg1_1956; Tables 3, 4).

**Table 4:** Comparison between overlapping sets of 17 outlier loci found by all three different methods^b^ of outlier analysis that compared the TOB_WILD population with the AQUA populations using Arlequin 3.5 (Figure 4). Outlier SNPs within 15 cM of the focal SNP from previous studies that included the SJR watershed. Candidate genes documented to affect growth, sexual maturity, or immune response within 5 Mb of the SNP (http://salmobase.org) are also shown

SNP NAME	Chr NA map^c^	NA^d^ map (cM)	Chr EU map^c^	EU^d^ map (cM)	Published outlier loci ± 15 cM EU female map	Candidate growth/gonad/immune genes within 5 Mbp of SNP
GCR_cBin4356_Ctg1_1956^k^^,^ m rs159407671	2^n^	–n	2	0.4		dcb (Near MHC class II antigen, CIGSSA_012441.t1); rapgef5 (Rap guanine nucleotide exchange factor, CIGSSA_010612.t1)
GCR_cBin25891_Ctg1_305^m^ rs159401753	3	96.9	3	83.2		Adjacent gene: StAR‐related lipid transfer protein 3|conserved (predicted) (CIGSSA_018950.t4); other genes: Igf2bp1 (insulin‐like growth factor 2 mRNA binding protein 1, CIGSSA_016974.t1); crhr1, corticotropin‐releasing hormone receptor 1, CIGSSA_016935.t1)
ESTNV_35352_77^e^^,^ m rs159407050	4	101.6	4	109.9		Adjacent gene: mlxipl or ChREBP (carbohydrate response element binding, (CIGSSA_023462.t1)
GCR_cBin37707_Ctg1_102^m^ rs159402156	5	43.7	5	47.8	EU_F 33.9 cM: 16466_1044^f^^,^ g; 66.9 cM: 17368_0088^f^^,^ g	Adjacent genes: CCR4‐NOT transcription complex subunit 6‐like and a disintegrin and metalloproteinase with thrombospondin motifs 2‐like
GCR_hBin33595_Ctg1_191^e^^,^ m rs159404016^a^	6	7.9	–o	–o		Adjacent gene: mitochondrial Rho GTPase 1 (CIGSSA_033844.t8)
ESTNV_17881_371^e^^,^ m rs159404117	9	96.2	9	91.9	EU_F 88.2 cM: ESTNV_34364_237^f^^,^ g	Immunoglobulin superfamily member 11 (CIGSSA_044008); unconventional myosin‐VIIa (CIGSSA_044110.t4)
ESTNV_24797_128^e^^,^ j ^,^ k ^,^ m rs159404243	12	38	12	68.7	EU_F 61.2 cM: GCR_cBin25404_Ctg1_420^i^	SNP is in a candidate gene (MHC class II beta chain); Adjacent gene: myosin heavy chain larval type 2 (CIGSSA_061303.t1)
ESTNV_16810_167^e^^,^ m rs159406247	12	70.4	12	108.3	EU_F 110.5 cM: 16129_0239^f^^,^ g EU_F105 cM: BASS113_B6A_E01_397^h^	Adjacent genes: ora1 (vomeronasal type‐1 receptor 4‐like) and ora2 (vomeronasal type‐1 receptor 1‐like); mhc‐sasa (MHC class II, class ii b, CIGSSA_063980.t1)
ESTNV_22997_260^e^^,^ m rs159404183^a^	13	52	13	85	NA_F 48.8 cM: 15806_943^g^	Adjacent genes: RILP‐like protein 1 isoform X1 and U11/U12 small nuclear ribonucleoprotein 35 kDa protein
GCR_cBin3895_Ctg1_167^m^ rs159402193	14	44.8	14	29.7		crh (corticotropin‐releasing hormone, CIGSSA_072826.t1); mhc1uxa2 (Mhc1uxa2 protein, CIGSSA_074346.t1)
ESTNV_29368_168^e^^,^ m rs159406544	15	34.9	15	44.2		bmp2 (bone morphogenetic protein 2, CIGSSA_080330.t1)
GCR_cBin15233_Ctg1_136_V2^i^^,^ k ^,^ m rs159408174^a^	15	53.4	–o	–o		Intron in mdag2 (MAM domain‐containing glycosylphosphatidylinositol anchor protein 2), TSHR (thyrotropin receptor, XM_014145015.1)
GCR_cBin2472_Ctg1_142^m^ rs159401697	15	59.6	15	77.2		Rab‐32 (Ras‐related protein, CIGSSA_078732.t1)
GCR_cBin27732_Ctg1_177^l^^,^ m rs159401836^a^	19	54.4	19	42		Adjacent genes: cadherin‐10‐like and cadherin‐6‐like; Mcr4 (Melanocortin receptor 4, CIGSSA_099455)
ESTV_14714_122^e^^,^ m rs119097051	19	61.5	19	52.7		ZupT (zinc transporter ZIP11‐like) contains SNP; sstr5 (somatostatin receptor 5 or growth hormone‐inhibiting hormone (GHIH) expressed in gut^j^
GCR_cBin6274_Ctg1_67^m^ rs159403881	20	29.9	20	34.3	EU 39.7 cM: 16260_0757^f^^,^ g	Rab‐35 (Ras‐related protein, CIGSSA_102343); gnrh1, (gonadotropin‐releasing hormone 1, XM_014160183.1), lysosome membrane protein 2‐like isoform X1, X2
ESTNV_31210_275^e^ rs159404664^a^	1p/23	118.1	23	51.4		myo5c (unconventional myosin‐Vc, CIGSSA_112000)

a *p* = .001 or smaller.

b Nonhierarchical AMOVA (Appendix S1), pairwise AMOVA (Appendix S3), and hierarchical AMOVA (Appendix S5).

c “Chr” means the chromosome number on which the outlier locus was located.

d “NA” means the position on the North American Atlantic salmon female linkage map by Brenna‐Hansen et al. (2012); “EU” means the position on the European Atlantic salmon female linkage map by Lien et al. (2011).

e See Table 3 for protein homology for this locus.

f Freamo et al. (2011).

g Culling et al. (2013).

h Mäkinen et al. (2014).

i Johnston et al. (2014).

j Very et al. (2008).

k Three of the 10 loci identified by BayeScan (Appendix S2).

l One of the three outlier loci was identified by all six pairwise comparisons between an AQUA population and the TOB_WILD population.

m Also significant for *F*_CT_ in the hierarchical Arlequin analysis.

n Chromosome containing SNP inferred from European map (Lien et al., 2011) after known translocations were accounted for (Brenna‐Hansen et al., 2012); consequently, the exact position on the chromosome is unknown.

o SNP has not been located on European map (Lien et al., 2011).

Many outlier loci found using all four methods were homologous to known proteins and could be located on the linkage maps and on the physical map of the Atlantic salmon genome (Table 3). Protein homologies were found for all 17 “consistent” outlier loci that had “EST” prefixes, indicating these SNPs were discovered by aligning expressed sequence tags (Table 3). Many outlier loci with a prefix of “GCR” (genome complexity reduction sequences) could not be identified by BLASTx, perhaps because the available DNA sequence containing the SNP was too short to conclusively identify a protein or because they were in noncoding regions.

### 3.5 Outlier detection—STW_WILD versus AQUA

The Method 1 nonhierarchical FDIST2 Arlequin analysis of the STW_WILD population and the six AQUA populations detected 54 outlier loci (Appendix S4) putatively under divergent selection. Just one of these (GCR_cBin8095_Ctg1_103) was also detected by both hierarchical and nonhierarchical Arlequin analyses of the AQUA populations with the TOB_WILD population (Appendices S1 and S5). This SNP was in the coding region of “protein phosphatase 1 regulatory subunit1B‐like” adjacent to the protein “stAR‐related lipid transfer protein 3‐like isoform X2” (Table 3). The results of the Method 1 nonhierarchical and the Method 3 hierarchical analyses of STW_WILD versus AQUA produced very similar *F*_ST_ outlier loci with the latter finding only one new *F*_ST_ outlier locus ESTNV_34538_503 (Appendices S4 and S6). On the other hand, the hierarchical Arlequin analysis (Table S2) found a total of 47 high‐*F*_CT_ outlier loci. Of these outlier loci, five were not significant (*p *< .01) for *F*_ST_ in either the nonhierarchical or hierarchical analyses and were therefore completely new outlier loci (Appendix S6). A highest number of significant high‐*F*_CT_ loci were clustered on chromosomes *Ssa*13, *Ssa*15, and *Ssa*19 (Appendix S6). Of the five outlier SNPs identified as being between 55.1 and 63.0 cM on *Ssa*19 of the NA female map, two in BASS120_B7_F09 were identified as being in the intron of Importin‐4 predicted protein located on the physical map at position 74.253203 to 74.272976 Mb. The minor allele of one of three outlier SNPs in ContigGCR_hBin7522_Ctg1 (rs159404032) was identified by BLASTN to be mRNA XM_014173899 (100% identity 5e‐17) for coiled‐coil domain‐containing protein 138 (ccdc138) which is located on the physical map at 28.524355 to 28.538390 Mb on *Ssa*25 (CIGSSA_117402).

### 3.6 Colocalization between outlier loci, QTLs, and candidate genes for production traits

Many of the 17 “consistent” outlier SNP loci from the AQUA versus TOB_WILD outlier analyses were on the same chromosome arm as candidate loci, or published QTLs for growth, maturity, or immune traits (Table 4). For example, the four outlier loci were in or located near candidate genes on the physical map that are part of the immune system. One outlier SNP locus was in a MHC gene (ESTNV_24797_128), ESTNV_16810_167 was near the candidate gene MHC class IIb and colocalized with previously published outliers, and two others (GCR_cBIN4356_Ctg1_1956 and ESTNV_17881_371) were near candidate loci that are part of the immune system (Table 4). Of even more interest were the three outlier loci located near candidate genes on the physical map associated with appetite and feeding. ESTNV_35352_77 was adjacent to a carbohydrate response element, GCR_cBin27732_Ctg1_177 was near melanocortin receptor 4, and ESTV_14714_122 was near somatostatin receptor 5 (Table 4).

Manhattan plots showed that some as highly significant *F*_CT_ outliers (on *Ssa*13, *Ssa*19 and *Ssa*23) did occur in the same regions of the linkage map as published QTL for growth and maturity (Figure 3C: j13, b14, c19, d19, b23) but that other *F*_CT_ outliers (on *Ssa*6) did not (Figure 3c). There was no overall correlation between the position of the outlier loci and SNP markers for published QTL for weight or maturity. The chi‐square test of association that compared the number of outlier loci and the number of QTL in each chromosome bin was not significant (*p* = 1.0). In addition, the Pearson correlation between the maximum −log_10_ *p* value of a QTL and the maximum −log_10_ *p* value of a *F*_CT_ outlier loci was not significant (Table S3). However, the correlation between the maximum −log_10_ *p* value of a QTL and the maximum number of studies that had detected it was significant (Table S3).

## 4 Discussion

### 4.1 Outlier loci can identify major locus traits for production traits

We hypothesized that fundamental questions about the genetic basis of economically important traits could be addressed by comparing allele frequencies at 3980 SNP loci between a farmed strain of Atlantic salmon established five generations ago and its wild founder population. We found a total of 17 “consistent” high‐*F*_ST_ outlier loci (Figure 4), three loci were in or near areas of the genome that contained candidate loci affecting appetite or metabolism and therefore potentially affecting growth, and four loci were in or near candidate genes conferring resistance to particular diseases or parasites (Table 4). Three of the most significant outlier loci were in chromosome regions containing published QTLs for growth or maturity. These results support our hypothesis that comparison of recently domesticated population with its wild founder population facilitates the discovery of candidate loci for traits under strong deliberate and accidental selection in the new hatchery environment. In dogs, phenotypes that vary most conspicuously among recently derived breeds, including size, limb length, coat color, coat texture, behavior, diet, skeletal morphology, and physiology have been used to identify the genomic regions that possess strong signatures of recent selection and contain major candidate genes (Akey et al., 2010; Axelsson et al., 2013; Pollinger et al., 2005; Von Holdt et al., 2010).

We qualitatively detected some outlier loci that colocalized with candidate loci and with QTLs for growth even though the overall associations between outlier loci and QTL were not significant. Since their independent establishment, the four independent AQUA populations (Table 1) have each experienced artificial selection for fast growth rate, and a low incidence of early sexual maturation beginning in the early 1980s (O'Flynn et al., 1999; Quinton et al., 2005; J. A. K. Elliott, unpublished data) and “accidental” domestication selection for adaptation to hatchery conditions since 1968. Genetic gains in quantitative traits such as weight at age are cumulative and have been shown to be measurable statistically in Atlantic salmon after only one generation of selection (Friars et al., 1995). Bourret, Dionne, and Bernatchez (2014) argued that natural selection on polygenic traits like survival in salmon would be difficult to detect with single outlier loci and proposed a multivariate solution. In turbot, partial colocalization was observed between candidate genes for growth and previously published QTLs, whereas high colocalization was observed for candidate genes associated with immune response and previously published QTLs for resistance to disease or parasites (Figueras et al., 2016). Simulations of traits determined by 1 to 10 QTL suggest that outlier “SNP”‐like loci physically linked to QTL are more likely to show colocalization with the nearest QTL than randomly chosen “SNP” loci but that false positives are common (Vilas et al., 2012).

Studies with livestock show that genome scans are more likely to detect signatures of selection for single locus traits, such as coat color, the absence of horns, or immune traits than for polygenic traits, such as milk yield and growth. This is true even in species, like cattle that have enormous pedigreed datasets from high‐density SNP chips, well‐annotated genomes, and precisely mapped QTLs for production traits (Kemper, Saxton, Bolormaa, Hayes, & Goddard, 2014; Stella, Ajmone‐Marsan, Lazzari, & Boettcher, 2010). In cattle and sheep, researchers have detected selection signatures associated with candidate genes for traits under deliberate artificial selection such as carcass yield, tail fat deposition, dairy traits (Moradi, Nejati‐Javaremi, Moradi‐Shahrbabak, Dodds, & Mce‐wan, 2012; Rothammer, Seichter, Forster, & Medugorac, 2013), reproductive traits (Gautier & Naves, 2011; Qanbari et al., 2011), coat color, and horn development (Druet, Pérez‐Pardal, Charlier, & Gautier, 2013) but also have detected signatures for immune responses (Gautier & Naves, 2011). In pigs, selection signatures have been identified in genomic regions associated with selected traits such as coat color, ear morphology, reproductive characteristics, and fat deposition (Wilkinson et al., 2013; adaptation to low temperatures (Ai et al., 2015) and also immune traits (Yang, Li, Li, Fan, & Tang, 2014)). In chickens, researchers have identified 82 selection signatures associated with traits under artificial selection such as eggshell hardness but have also detected those associated with immune system characteristics (Qanbari et al., 2012).

### 4.2 Outliers in similar genome regions among studies?

Our study found a different and nonoverlapping set of SNP outlier loci than that found by two previous studies that compared similar wild and aquaculture strains of North American Atlantic salmon from the SJR system. Vasemägi et al. (2012) found four outlier loci of a 320 SNP dataset consisting of six fish from the Nashwaak tributary of the SJR and six from the 2003 SGRP (Salmon Genetics Research Program) year class SJR aquaculture strain (Quinton et al., 2005). All four of his outlier loci were also successfully genotyped by Mäkinen et al. (2015) and by our study but were not identified as outliers in either case. On the other hand, some of the outlier loci that differ between studies are in the same region of the genome. For example, the outlier BASS113_B6A_E01_397 that was found by Mäkinen et al. (2015) is within 3 cM of one of our consistent outlier SNPs (ESTNV_16810_167, Table 4) that is located on chromosome *Ssa*12.

We identified about 1% of the SNPs we genotyped as outlier loci even after FDR was considered. This is similar to previous studies of wild fish populations that typically identify between 1% and 10% of the loci surveyed as outlier loci (Bradbury et al., 2013; Narum et al., 2010). We recognize that not all our outlier loci are necessarily a result of differences in local extrinsic selection. Recent theoretical work has shown that various factors other than selection can influence heterogeneous divergence of genomic regions among populations including demographic history, geographic structure, and close physical linkage (Klopfstein, Currat, & Excoffier, 2006).

As expected, all AQUA populations were characterized by smaller genetic distances from the TOB_WILD population than from the STW_WILD population, supporting the hypothesis that the TOB_WILD was the most closely related to the AQUA founder population. AQUA2009 was most distantly related to the other AQUA populations likely because it consisted only of the eight full‐sibling “multiplier” families and their parents which would be expected to modify the allele frequencies from what they would have been had all the parents of the 2009 year class been sampled (Hansen, Nielsen, & Mensberg, 1997).

### 4.3 Correlation between outlier loci and major QTL for sea age

Although comparisons of closely related populations are usually most useful for detecting outlier loci under diversifying selection (Li et al., 2013), the analysis of STW_WILD versus AQUA identified an outlier locus correlated with a major QTL associated with age at sexual maturity. STW_WILD is an iBoF population that has more one‐sea winter spawners (grilse), whereas TOB_WILD is an oBoF population that has more multisea winter spawners (P. O'Reilly, pers. comm.). Our hierarchical Arlequin analysis of STW_WILD versus AQUA identified three outlier SNPs within ContigGCR_hBin7522_Ctg1 (*Ssa*25: 28.53 Mb) downstream from a genomic region associated with sea age in wild salmon populations (*Ssa*25: 28.58 to 28.75 for males in Ayllon et al. (2015); *Ssa*25: 28.64 to 28.78 Mb for males and females in Barson et al. (2015)). The gene “ccdc138” containing these three SNPs was near the “sea age” at maturity region only 0.0386 Mb downstream from the associated locus “chmp2b” and only 0.116474 Mb downstream from the candidate gene “vgll3”.

In addition, our hierarchical Arlequin analysis of TOB_WILD versus AQUA identified an outlier locus in a similar position (ESTNV_34703_1491 on *Ssa*09: 27.5 Mb and 52.7 cM female map) to one previously associated with sea age in wild Atlantic salmon populations (Johnston et al., 2014) that was not significant in a second GWAS after accounting for population structure (Barson et al., 2015). It also identified a consistent outlier GCR_cBin15233_Ctg1_136_V2 on *Ssa*15 near the TSHR (the thyrotropin receptor) (Table 4). TSHR induces final sexual maturity in salmonids and is considered analogous to LH (luteinizing hormone) in vertebrates (Oba, Hirai, Yoshiura, Kobayashi, & Nagahama, 2001). Genetic changes that resulted in delayed binding of thyrotropin to the receptor (Oba et al., 2001) could have been selected for under strong artificial selection for delayed sexual maturity experienced by the AQUA strain.

### 4.4 Admixture removal before outlier detection

Search for outlier loci in North American populations without first removing fish with putative European ancestry could have led to the detection of a large number of false‐positive outlier loci in the analysis because of large differences in average allele frequencies at the loci on the same 6K SNP chip (Bourret, Kent, et al., 2013) that was used here. We believe that the well‐documented use of fish with European ancestry in salmon breeding populations in Maine, just south of the Bay of Fundy region where SGRP was developed (Glebe, 1998) justifies the use of our *K* = 2 (Fig. S1) STRUCTURE results (Fig. S2) to remove individuals with more than 5% European ancestry from the subsequent genetic distance and outlier analyses (Fig. S2). We acknowledge that, because of the small sample size of known pure and hybrid European fish included in our original STRUCTURE analysis, we were initially not confident that all of the 252 excluded fish had European ancestry. We also acknowledge that variation in our population sample size, which ranged from 8 to 268 for different populations in this study (Table 1), could have affected the STRUCTURE results. Fortunately, our subsequent unpublished STRUCTURE analyses of the dataset presented here combined with a larger North American and European dataset confirm our original interpretation.

### 4.5 Gene ontology of outlier loci and nearby candidate genes

The historical objective of the selection program for Atlantic salmon in the SJR has been rapid growth rate and late sexual maturity (Quinton et al., 2005; Saunders, 1981). Direct selection for polygenic traits, such as growth rate and late maturation, may result in indirect selection for a wide variety of biological pathways, including increased appetite and immune responses to specific pathogens or metabolism.

Strong selection for high growth rates could select for fish that spend more time eating. This mechanism could be responsible for the allele frequency differences between the AQUA and founder population at the consistent outlier GCR_cBin27732_Ctg1_177 which is located near melanocortin receptor 4 (Table 4). Melanocortin peptides affect motivation to feed in rainbow trout (Schjolden, Schiöth, Larhammar, Winberg, & Larson, 2009) and have previously found to be associated with weight gain and feed intake in pigs (Fontanesi et al., 2015). Two other consistent outlier loci are located near candidate genes associated with growth and metabolism. ESTV_14714_122 is located near somatostatin receptor 5 (Table 4). High levels of somatostatin are known to reduce growth rates in rainbow trout by lowering plasma levels of growth hormone (GH), insulin‐like growth factor‐1 (IGF‐I), and insulin (Very, Kittilson, Klein, & Sheridan, 2008). Alleles that reduce levels of somatostatin might therefore be indirectly selected for under strong artificial selection for increased growth rates. ESTNV_35352_77 is adjacent to a carbohydrate response element transcription factor that regulates carbohydrate metabolism in the liver and pancreatic β‐cells of humans in response to high levels of glucose (Noordeen et al., 2010). Selection for high growth rates on a high carbohydrate feed diet could result in genetic changes to that have affected the expression levels of this transcription factor. Taken together, these changes at multiple outlier loci may result in more efficient feed utilization of the SJR AQUA strain. Similar changes may explain the higher growth rate of a line of Atlantic salmon selected for fast growth relative to wild salmon despite its lower feed consumption (Thodesen, Grisdale‐Helland, Helland, & Gjerde, 1999).

Several outlier loci detected here are in (ESTNV_24797_128, Table 3) or near MHC and immunoglobulin proteins (ESTNV_16810_167, GCR_cBIN4356_Ctg1_1956, and ESTNV_17881_371) known to be involved in disease resistance (Gómez, Conejeros, Consuegra, & Marshall, 2011; Harstad, Lukacs, Bakke, & Grimholt, 2008). Allele frequency changes between the founder and AQUA populations may have occurred because of accidental selection that occurred when “ranched” wild fish were spawned at high densities in hatcheries (Fleming, Agustsson, Finstad, Johnsson, & Bjönsson, 2002; Tonteri, Vasemägi, Lumme, & Primmer, 2010) after the building of the Mactaquac hatchery on the SJR. There was never any deliberate artificial selection for disease resistance as part of the SGRP breeding program (Quinton et al., 2005) that could account for these changes but there may have been accidental selection.

### 4.6 Practical applications of identifying SNPs associated with growth and immune traits

This study detected two parallel and 17 consistently detected high‐*F*_ST_ outlier SNP loci between the AQUA and TOB_WILD populations many of which were associated with candidate genes for production traits. We are planning to incorporate SNPs in candidate genes for growth (somatostatin receptor 5), appetite (melanocortin receptor 4; carbohydrate response elements), sea age (vgll3; TSHR), and immune traits (MHC class2) into low‐density SNP assays so that the putative relationships with economically important traits can be validated in larger populations. The density of the 6K chip used here was too low to effectively detect fine‐scale patterns of among population divergence and within population homozygosity in response to population‐specific directional selection. More precise detection of long haplotype blocks from selective sweeps is now possible using the 220K SNP chip (Yáñez et al., 2016) and the high‐density 132K SNP chip (Houston et al., 2014) that have been developed for European Atlantic salmon.

To distinguish between linkages with causal genes and showing that a causal gene determines a quantitative trait, future eQTL work investigating the regulation of expression of candidate genes near the outlier loci discovered here might reveal their functional role in biological pathways. In this way, it may be possible to systematically elucidate how divergent selection between wild and farmed populations works on economically important quantitative traits. Relationships between the outlier SNPs and published QTLs will continue to be tested in more precise QTL‐mapping and GWAS studies using higher density SNP chips. Such studies would facilitate the application of modern marker‐assisted selection for the genetic improvement in Atlantic salmon stocks (e.g., Moen & Ødegård, 2014). Ultimately, a deeper understanding of artificial selection in Atlantic salmon and other recently domesticated species may inform how the genetic architecture of quantitative traits affects the mechanisms of evolutionary change in natural populations.

## Data Archiving Statement

Data available from the Dryad Digital Repository: http://dx.doi.org/10.5061/dryad.53b58.
