# Supplementary figures and images for: A genome scan for selection signatures comparing farmed Atlantic salmon with two wild populations: Testing colocalization among outlier markers, candidate genes, and quantitative trait loci for production traits
Source: Evol Appl. 2016 Dec 29;10(3):276–96. doi: 10.1111/eva.12450 (PMC5322405; doi:10.1111/eva.12450)

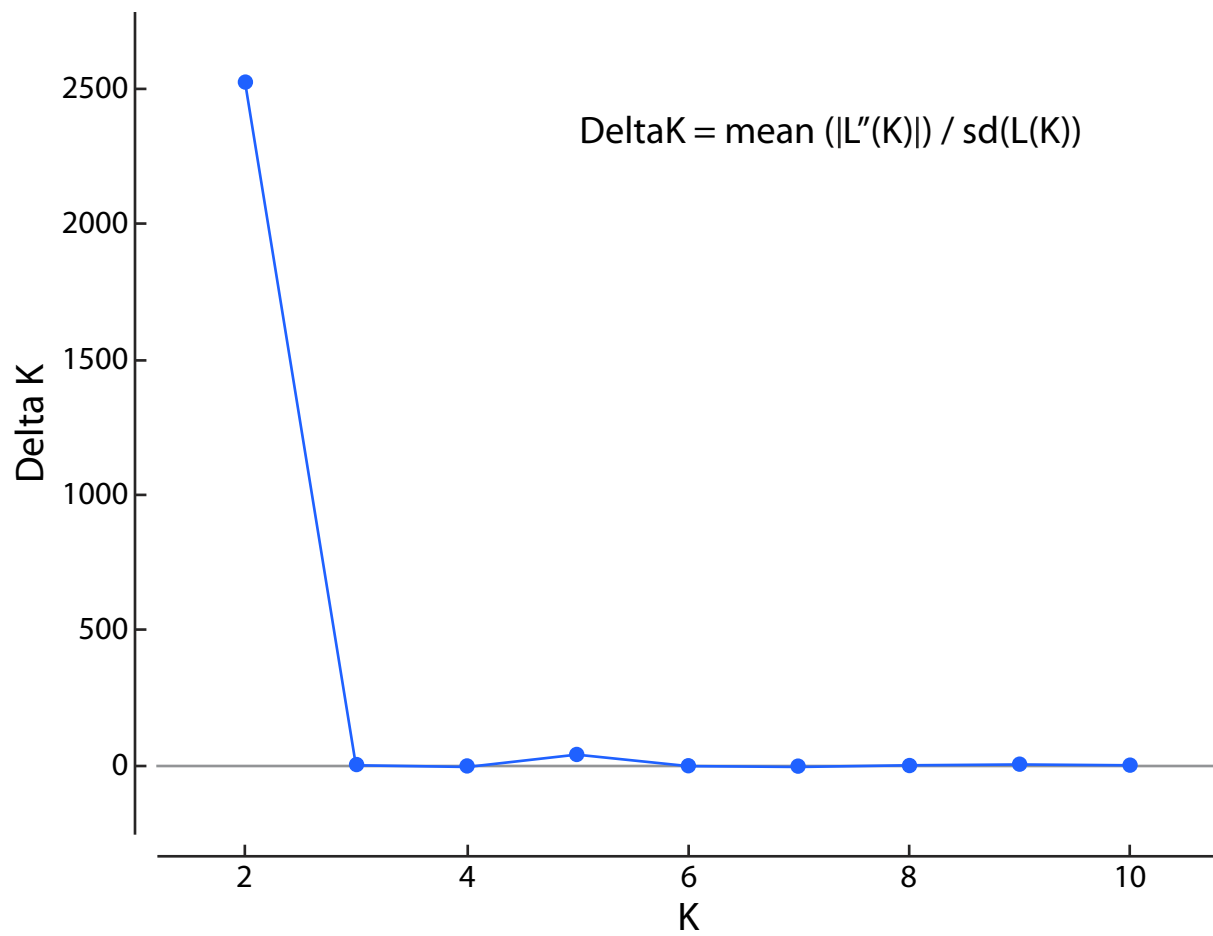

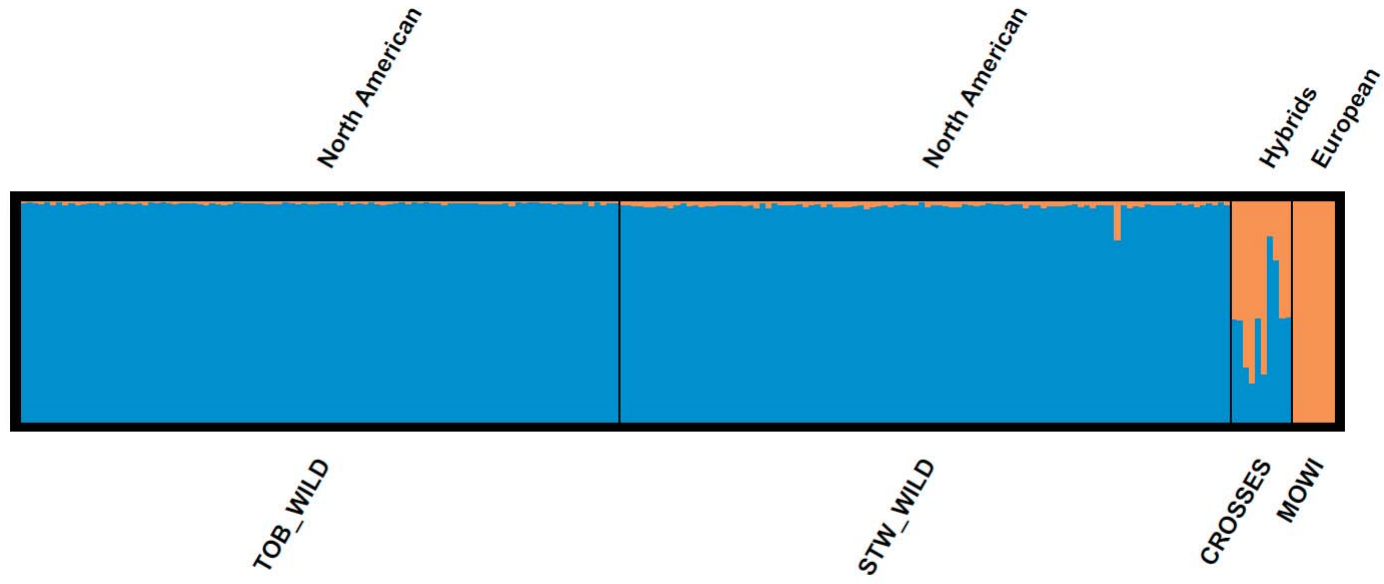

Supplement: Supplementary file 1 [file EVA-10-276-s001.pdf]
