# Supplementary material for: A genome scan for selection signatures comparing farmed Atlantic salmon with two wild populations: Testing colocalization among outlier markers, candidate genes, and quantitative trait loci for production traits
Source: Evol Appl. 2016 Dec 29;10(3):276–96. doi: 10.1111/eva.12450 (PMC5322405; doi:10.1111/eva.12450)
Supplement: Supplementary file 2 [file EVA-10-276-s002.docx]

**Supporting Information**

Additional Supporting Information that will be included in the online version of this article follows:

**Supplementary Tables**

**Table S1.** Locus-by-locus AMOVA from Arlequin 3.5 for 3980 loci with one group containing the randomly split TOB_WILD population and the other group containing the AQUA populations. Average over all loci: F_ST_ = 0.0266, F_SC_ = 0.0208, F_CT_ = 0.0059.

| Source of variation | Sum of squares | Variance components | Percentage variation |
| --- | --- | --- | --- |
| Among groups | 1800.51 | 1.779 | 0.59^**^ |
| Among populations within groups | 6345.37 | 6.192 | 2.07^**^ |
| Within populations | 416579.66 | 291.518 | 97.4 |
| Total | 424725.53 | 299.49 |  |

***P* < 0.00001.

**Table S2.** Locus-by-locus AMOVA from Arlequin 3.5 for 3980 loci using Method 3 with one group containing the randomly split STW_WILD population and the other group containing the AQUA populations. Average over all loci: F_ST_ = 0.0972, F_SC_ = 0.0208, F_CT_ = 0.078.

| Source of variation | Sum of squares | Variance components | Percentage variation |
| --- | --- | --- | --- |
| Among groups | 9899.85 | 25.215 | 7.8^**^ |
| Among populations within groups | 6371.51 | 6.207 | 1.92^**^ |
| Within populations | 418144.87 | 291.797 | 90.3 |
| Total | 434416.23 | 323.218 |  |

***P* < 0.00001.

**Table S3.** Pearson Correlation, r, for maximum values of four variables on each of 28 chromosomes (lower triangular matrix) and associated uncorrected P-values (upper triangular matrix). Variables are −log10 (P-value) for F_ST_, −log10 (P-value) for F_CT_, −log10 (P-value) for a QTL, and the number of QTLs found over 10 studies (see Methods text).

|  | −LOG_10_F_ST_ | −LOG_10_F_CT_ | −LOG_10_MINPQTL | COUNTQTL |
| --- | --- | --- | --- | --- |
| −LOG_10_FST |  | 0.046* | 0.778 | 0.328 |
| −LOG_10_F_CT_ | **0.380** |  | 0.209 | 0.452 |
| −LOG_10_MINPQTL | 0.056 | 0.245 |  | 0.033* |
| COUNTQTL | 0.192 | 0.148 | **0.403** |  |

**Supplementary Figures:**

**Fig. S1.** Evanno et al. (2005) plots for detecting the number of K groups that best fit the data. The modal value of this distribution is the true K(*) or the uppermost level of structure, suggesting here that the correct number of clusters is two.

**Fig. S2.** Estimated population structure for K = 2 “clusters” with the samples grouped and labelled by Atlantic salmon source population (Table 1). Each individual fish is represented by a thin histogram bar filled with either blue of orange that indicates cluster membership. Some “admixed” bars are filled with almost equal amounts of blue and orange fill segments, which correspond to their genome’s membership coefficients belonging equally to both clusters. Each color in Figure 2S represents a different cluster, and each individual fish is indicated by a bar of the histogram. The putative populations are separated by vertical black lines. The majority individuals of in the putative North American wild population had a high probability of belonging to the first cluster, and are therefore represented by a bar filled with a blue shading. In contrast, all individuals from the documented F1-hybrids and the pure Mowi European family had a high probability of belonging to a second cluster, and are represented by a bar filled with orange shading. The detailed STRUCTURE analysis suggested that some individuals in the AQUA populations had ancestry from both North American and European subspecies. These individuals were removed from the dataset before subsequent outlier analysis.

**Legends for Supplementary Appendices in the Microsoft Excel file**

**Appendix S1.** Nonhierarchical Arlequin 3.5 analysis of five different groups of the SJR Aquaculture population [2009PO_AQUA, 2010PG_AQUA, 2011PG_AQUA, 2011PGN_AQUA, 2012PG_AQUA (Table 1)] as well as a sample from the wild Tobique River population (TOB_WILD). Column A "SNP number" refers to the input order of the loci in the data file and was used to label the points in Figure 1A. Column B "Index number" and Column C “SNP name” match those on the CIGENE 6K SNP chip. Column D “P-values” and Column E “FST values” show the analysis results. Column F shows q-values that give FDR from SGoF plus method. Columns G to J show the North American map positions for the corresponding dbSNP reference cluster number (Brenna-Hansen et al. 2012) and Columns J to M show the European subspecies map positions for the corresponding dbSNP reference cluster number (Lien et al. 2011). “n/a” is “not applicable” because this SNP could not be mapped. The 37 high FST loci outliers at the 1% level sorted by decreasing FST values are displayed in red font. The results for the remainder of the 3980 loci are shown below in black font.

**Appendix S2.**  Bayescan analysis of five different groups of the SJR Aquaculture population [2009PO_AQUA, 2010PG_AQUA, 2011PG_AQUA, 2011PGN_AQUA, 2012PG_AQUA (Table 1)] as well as a sample from the wild Tobique River population (TOB_WILD). Column A "SNP number" refers to the input order of the loci in the data file and was used to label the points in Figure 1B. Column B "Index number" and Column C “SNP name” match those on the CIGENE 6K SNP chip. Column D “q-values” and Column E “FST values” show the analysis results from BayeScan. Columns F to I show the North American map positions for the corresponding dbSNP reference cluster number (Brenna-Hansen et al. 2012) and Columns J to M show the European subspecies map positions for the corresponding dbSNP reference cluster number (Lien et al. 2011). “n/a” is “not applicable” because this SNP could not be mapped. The 10 high FST outlier loci with a q-value less than 0.05 sorted by decreasing FST values are displayed in red font. The results for the remainder of the 3980 loci used in the analysis are shown below in black font.

**Appendix S3.**  Appendix S3. Pairwise FDIST2 Arlequin 3.5 analyses results showing outlier loci found for all pairwise comparisons of one of the six different groups of the SJR Aquaculture strain with the wild Tobique population (see also Figure 2). Column A "SNP number" refers to the input order of the loci in the data file and was used to label the points in Figure 1B. Column B "Index number" and Column C “SNP name” match those on the CIGENE 6K SNP chip. Columns D to U show the pairwise analysis results. Columns V to Y compare with other analyses. Columns Z to AC show the North American map positions for the corresponding dbSNP reference cluster number (Brenna-Hansen et al. 2012) and Columns AD to AG show the European subspecies map positions for the corresponding dbSNP reference cluster number (Lien et al. 2011). “n/a” is “not applicable” because this SNP could not be mapped. Columns AH to AJ compare with previous outlier loci studies. Columns AK to AL give protein and gene ontology for SNPs. A total of 132 high FST outlier loci significant at the 1% level in at least one pairwise comparison are listed alphabetically with the significant P-values and the corresponding FST values shown in red font. The results for the other loci are not shown.

**Appendix S4.** Nonhierarchical FDIST2 Arlequin 3.5 analysis of five different groups of the SJR Aquaculture strain [2009PO_AQUA, 2010PG_AQUA, 2011PG_AQUA, 2011PGN_AQUA, 2012PG_AQUA (Table 1)] as well as a sample from the wild Stewiacke population (STW_WILD). Column A "SNP number" refers to the input order of the loci in the data file. Column B "Index number" and Column C “SNP name” match those on the CIGENE 6K SNP chip. Column D “P-values” and Column E “FST values” show the analysis results. Column F shows q-values that give FDR from SGoF plus method. Columns G to J show the North American map positions for the corresponding dbSNP reference cluster number (Brenna-Hansen et al. 2012) and Columns K to N show the European subspecies map positions for the corresponding dbSNP reference cluster number (Lien et al. 2011). “n/a” is “not applicable” because this SNP could not be mapped. The high FST outlier loci significant at the 1% level are displayed in red font from largest to smallest. Column O shows that only one outlier SNP was also a high FST outlier SNP when the same analysis was performed on the TOB population.

**Appendix S5.** Appendix S5. Hierarchical Arlequin analysis with the three large populations of the SJR Aquaculture strain in one group [2010PG_AQUA, (2011PG_AQUA + 2011PGN_AQUA), 2012PG_AQUA] and the split wild Tobique population in the other group (TOB_WILD). Column A "SNP number" refers to the input order of the loci in the data file and was used to label the points in Figure 1A. Column B "Index number" and Column C “SNP name” match those on the CIGENE 6K SNP chip. Columns D to J show the hierarchical Arlequin outlier analysis results (Columns F and J shows q-values that give FDR from SGoF plus method). Column K shows SNPs that were outliers in the non-hierarchical analysis. Column L shows loci that were outliers in the Bayescan analysis. Column M shows SNPs that were outliers in the pairwise analysis. Column N shows 16 SNPs that were consistently outliers in all three Arlequin analyses with the TOB population to the AQUA population. Columns O to R show the North American map positions for the corresponding dbSNP reference cluster number (Brenna-Hansen et al. 2012) and Columns S to V show the European subspecies map positions for the corresponding dbSNP reference cluster number (Lien et al. 2011) with “not applicable” abbreviated as“n/a” because this SNP could not be mapped. Column W shows FCT outliers for the Stewiacke analysis. Column X shows two FCT outliers that were also found by Bourret (2013a). Columns Y and Z show that none of these outliers were FCT North American or intercontinentaloutliers found by Bourret(2013b). Column Z lists protein homologies shown in Table 3. Column AA lists protein homologies found by Bourret (2013a) including those for the 11 loci for which FST was not significant in the non-hierarchical Arlequin analysis and were therefore not given in Table 3. Results are sorted by decreasing FCT value. FCT outlier loci that were outliers at the 1% level for FST in the non-hierarchical Arlequin analysis are displayed in red font.

**Appendix S6.** Hierarchical Arlequin analysis with the three large populations of the SJR Aquaculture strain in one group [2010PG_AQUA, (2011PG_AQUA + 2011PGN_AQUA), 2012PG_AQUA] and the split wild Stewiacke population in the other group (STW_WILD). Column A "SNP number" refers to the input order of the loci in the data file and was used to label the points in Figure 1A. Column B "Index number" and Column C “SNP name” match those on the CIGENE 6K SNP chip. Columns D to J show the hierarchical Arlequin outlier analysis results (Columns F and J shows q-values that give FDR from SGoF plus method). Column K shows SNPs that were FCT outliers in the TOB hierarchical analysis. Column L shows loci that were outliers in the TOB Bayescan analysis. Column M shows SNPs that were outliers in the TOB pairwise analysis. Column N shows 16 SNPs that were consistently outliers in all three Arlequin analyses with the TOB population to the AQUA population. Columns O to R show the North American map positions for the corresponding dbSNP reference cluster number (Brenna-Hansen et al. 2012) and Columns S to V show the European subspecies map positions for the corresponding dbSNP reference cluster number (Lien et al. 2011) with “not applicable” abbreviated as “n/a” because this SNP could not be mapped. The FCT outlier loci for which FST was also significant at the 1% level are displayed in bold font. Column W shows that only one outlier SNP was also a high FCT outlier SNP when the same analysis was performed on the TOB population.
